# Hospitals under 150 Beds

**Published:** 1916-12-16

**Authors:** 


### FURTHER HOSPITAL APPEALS.

### Hospitals under 150 Beds.

**CHELSEA HOSPITAL FOR WOMEN.**—The new hospital in Arthur Street, Chelsea, which was opened by H.M. the Queen in July last, has now been in occupation four months, greatly to the advantage of the patients. As soon as the new Nurses' Home can be built that part of the hospital which is being used temporarily for the nursing and domestic staffs will be vacated, and twenty-three more beds will be available for patients. This completion of the rebuilding scheme will have a marked effect on the long "waiting list," and improve the special facilities for admission which are being given to relatives of our sailors and soldiers. A sum of £25,000 is still required. The Convalescent Home at St. Leonards-on-Sea continues to perform its invaluable work for patients recovering from severe operations, as well as for those sent independently by subscribers. Donations and new subscriptions will be most gratefully received by the Hon. Treasurer, Mr. Sidney H. Goldsmid, or by the Secretary, Mr. Herbert H. Jennings, at the hospital.

**CITY OF LONDON LYING-IN HOSPITAL.**—The work which this hospital performs, in preserving the infant life of the community and caring for the mothers at the critical time of childbirth, is now, more than ever before, one of the most important problems of the day, and the nature of it must appeal strongly to the human side of everyone. There is still £6,000 outstanding of a loan for the rebuilding of the hospital, which became imperative when the erection of a Tube station opposite caused serious damage to the foundations of the old building in 1907. It is also estimated that there will be a deficit of £1,500 at the end of the year. To maintain the service of the hospital at its present high state of efficiency it is absolutely essential that it receive greater support from the public. Secretary, Mr. E. Lionel Brown.

**EAST LONDON HOSPITAL FOR CHILDREN, SHADWELL, E.**—The hospital stands in urgent need of help. The economy of its management has been commended by those who control the King Edward's Hospital Fund, and further reduction in current expenses is impossible. Therefore if no help comes it will have to close its doors. This is unthinkable in these times, when the care of the children of our fighting men is an obligation laid upon us all, and the health of the new generation is more than ever of vital importance to the nation. Gifts of money, great or small, will be gratefully received by the Secretary, Mr. W. M. Wilcox.

**EVELINA HOSPITAL FOR SICK CHILDREN.**—The charity is urgently in need of increased support. Owing to its position it is seldom seen, and suffers accordingly, although it is carrying on as good a work as any charity in London; it has, therefore, special claims upon the benevolent public, and it is sincerely hoped, notwithstanding extra appeals now being made, that its needs will not be overlooked. Contributions are sorely needed, as, owing

### FURTHER HOSPITAL APPEALS.

### Hospitals under 150 Beds.

to the heavy rise in the cost of provisions and other necessaries, the general expenditure of the institution has largely increased; whilst, owing to the war, contributions have fallen off considerably. Unless substantial financial help is soon forthcoming it may become necessary to close some of the wards. Contributions will be gratefully received by the Secretary, Mr. H. C. Staniland Smith.

**HOSPITAL FOR EPILEPSY AND PARALYSIS.**—"The tragedy of shattered nerves is more awful than that of a maimed or even destroyed body." This text is illustrative of the work being done at the hospital during the past half-century. The hospital has a military section of thirty-five beds for soldiers suffering from shattered nerves. It is under agreement to provide beds for twenty-five sailors at twenty-four hours' notice. Fortunately it has not been necessary to use the accommodation as yet, and the twenty-five beds, together with thirty specially reserved for civilians, making fifty-five in all, have been constantly occupied by the wives and children of soldiers. Contributions towards the £8,000 requisite for carrying on the hospital's work are earnestly solicited. Mr. H. W. Burleigh is the Secretary.

**MOUNT VERNON HOSPITAL FOR CONSUMPTION, NORTHWOOD, MIDDLESEX,** is in great need of annual subscriptions and donations. Owing to the increased cost of maintenance, the expenditure is in excess of the income for the current year. The treatment of women and children is a special feature. Mr. W. J. Morton, Secretary.

**NATIONAL HOSPITAL FOR DISEASES OF THE HEART, WESTMORELAND STREET, W.**—This is the only institution of its kind in the United Kingdom, being the sole special hospital devoting its work to the alleviation of those suffering from diseases of the heart. A debt of £9,000 causes much anxiety, and contributions directed to the Secretary, Mr. Robert G. E. Whitney, will be gratefully received.

**POPLAR HOSPITAL FOR ACCIDENTS.**—The hospital issues a leaflet containing reasons for helping, amongst which is the fact that the institution is situated in the centre of the Port of London, and is in the best possible position for dealing with the serious accidents—owing to the war pressure, more numerous and serious than ever—which occur at the docks and at the hundreds of factories in the East End of London. One night alone twenty-two victims of a Zeppelin raid were brought in. The number of beds has been increased to 117, including twenty-four for wounded soldiers. Over 1,100 wounded men since December 1914 have been treated as in- and out-patients. The hospital has never been in debt, but though out of debt is not out of date. Funds for maintenance are asked for. Mr. Percy Rogers is the Secretary.

**PRINCE OF WALES'S GENERAL HOSPITAL, TOTTENHAM.**—Much difficulty has been experienced in raising the £12,000 needed annually for maintenance, and there is a debt of £8,000. No letters are required at this hospital, while 30,000 patients are treated each year. Mr. Fredk. W. Drewett is the Secretary.

**QUEEN CHARLOTTE'S LYING-IN HOSPITAL, MARYLEBONE ROAD, N.W.**—The demands on the assistance of Queen Charlotte's Lying-in Hospital, Marylebone, during the present year have been larger than ever before, owing to the fact that the hospital is admitting a great number of the wives of our sailors and soldiers as well as Belgian and other refugees. Since the outbreak of war over 3,000 wives of our sailors and soldiers have either been received into the hospital or attended in their own homes. Any reduction in the number of beds for patients would be little short of a calamity, yet it is difficult to see how the hospital can continue its work on the present scale unless substantial additional support is forthcoming. There is at present a debt of about £5,000 on the General Maintenance Account. The hospital has just opened a new (temporary) building adjoining the hospital for the ante-natal department, where this most important work will be carried on under more favourable conditions than formerly. An Infant Consultation Centre is also being established. When so many lives are being laid down in the service of the country it is more than ever necessary to save the children, and Queen Charlotte's Hospital deserves the liberal support of the public to enable it to carry on satisfactorily its increasing and valuable work. Mr. Arthur Watts is Secretary.

**QUEEN'S HOSPITAL FOR CHILDREN, KINGSLAND ROAD, N.**—The calls upon this institution have been steadily augmented with the increase of the working classes in Hackney. A further strain has recently been placed upon its resources by the results of the restrictions necessarily placed on the reception of children at the General Hospitals and Poor-Law Infirmaries, which are treating large numbers of soldiers. The expenditure of the hospital and its Bexhill Home is in normal times about £16,500 a year, but under present circumstances £20,000 has to be raised annually for maintenance purposes, towards which only £1,000 is obtained from other than voluntary sources. At the present time a sum of £2,500 is needed to enable the year to be closed without serious difficulties. Annually about 2,000 children are treated in the wards and 40,000, making 90,000 attendances, in the out-patients' department.

**ROYAL HOSPITAL FOR DISEASES OF THE CHEST, CITY ROAD.**—Lord Hollenden, the President, urges that it would be nothing short of a calamity affecting the dignity of the nation if this hospital, under the patronage of our beloved Sovereign, should close for want of funds, and the more so should this happen after its splendid work for over a century, and at a time, too, when the need for its work is at the greatest. Every donation will now help to heal sick and wounded from the battlefields; it will also prepare the hospital for its equally great work after the war.

**ST. MARK'S HOSPITAL, CITY ROAD.**—The greatest need at presnt is the help of the charitable public in securing the necessary funds to continue our work in curing and relieving a large number of cancer and other rectal cases. The increased prices on all articles of food, etc., has naturally increased the expenditure, and as the war continues this difficulty will, of course, be greater. Therefore increased generous help is asked for. The Secretary is Mr. H. W. G. Coope.

**SAMARITAN FREE HOSPITAL FOR WOMEN, MARYLEBONE ROAD, N.W.**—The committee of management of the Samaritan Free Hospital for Women, the only entirely free hospital for women in London, appeal for Christmas benefactions to enable them to meet the outstanding tradesmen's accounts amounting to £1,250 and to reduce the bankers' loan of £1,500. Special contributions are earnestly solicited towards the endowment of a ward for cancer, of patients suffering from which

### FURTHER HOSPITAL APPEALS.

### Hospitals under 150 Beds.

disease upwards of 100 cases were treated last year, and many are waiting admission. The committee hope that by instituting a special ward for these poor sufferers much urgently needed relief will be forthcoming. Mr. George Hawkins, Secretary.

**SOUTH LONDON HOSPITAL FOR WOMEN.**—This new hospital was opened by the Queen on July 4 last, has eighty beds for women and children, and is entirely officered by medical women. A special feature is the provision of sixteen beds in private wards for women of limited means at low inclusive fees. The increasing numbers of women and children attending the out-patient department at 88-90 Newington Causeway have necessitated an extension of the premises, and a special fund is being raised to meet the cost of renting a third adjoining house and adapting it to meet the purpose for which it is required. Owing to the steady rise in the price of almost all hospital requisites, the cost of equipping the new building has been far greater than was anticipated. On the other hand, the numerous claims upon the public arising out of the war have made it unusually difficult to obtain support for this new institution. The board of management earnestly appeal for contributions and promises of help, which will be gladly received by the Secretary of the Hospital, South Side, Clapham Common, S.W.

**VICTORIA HOSPITAL FOR CHILDREN, CHELSEA, S.W.**—During the last two years the annual subscriptions have decreased to the extent of over £300, and the difficulty in realising estates impedes the payment of legacies, on which it is relied to prevent a deficit on the working expenses at the end of the year. The committee have drawn nearly all their small reserve fund, and there is immediate need of £1,000 to make both ends meet at the end of the year. The Secretary is Mr. H. G. Evered.

